# The rights (and responsibilities) of the public to advance health through research

**DOI:** 10.1186/s13690-021-00726-w

**Published:** 2021-11-16

**Authors:** Fabrice Jotterand, Ryan Spellecy, Reza Shaker

**Affiliations:** 1 Center for Bioethics and Medical Humanities, Medical College of Wisconsin, 8701 Watertown Plank Road, WI 53226 Milwaukee, USA; 2 Institute for Biomedical Ethics, University of Basel, Bernoullistrasse 28, 4056 Basel, Switzerland; 3 Division of Gastroenterology and Hepatology, School of Medicine, Center for Translational Science Institute of Southeast Wisconsin, Medical College of Wisconsin, 8701 Watertown Plank Road, WI 53226 Milwaukee, USA

**Keywords:** Public, Rights and responsibility, Research

## Abstract

In this commentary we assert that the rights and responsibilities of the community at large as an important key stakeholder in the effort of advancing health through research and discovery have not been carefully examined and delineated. The time has come to investigate the nature of the rights and responsibilities of the public in advancing health through research and discovery. We argue that the public has the right not merely to participate in research and have their rights protected, but that they have a right to engage in and take ownership in research.

## Background

Sir William Osler (1849-1919) notes that “the good physician treats the disease. The great physician treats the patient with the disease”. A *good* physician, Osler points out, is an individual who addresses the root cause of a physical ailment (the disease) whereas the *great* physician also considers the environment in which a disease manifests itself: the patient and his/her milieu. In the context of biomedical research, the same truth holds: biomedical research should not only address specific diseases but also focus on the broader social context in which scientific inquiry and discovery takes place and, expanding beyond Osler’s considerations of the physician and patient, consider carefully who the stakeholders are in this effort. Broadly speaking, biomedical research aims at improving health and entails the involvement of two major groups: the research community and the public. Thus far we have made *good* progress in advancing health through research and discovery by empowering researchers and clinicians to engage in biomedical research and delineating their duties and responsibilities. Some of the rights and responsibilities of the research community in pursuing their goals has been well defined and safeguarded by the principles established by the Belmont Report. [1] The Report, however, does not consider carefully the role of the public as the owner and steward of the research enterprise. *Great* progress in advancing health through research and discovery requires a paradigm shift that started early 2000s.

We take it as a given that the public has rights and responsibilities in advancing health through research and discovery, as they are the main funders of research through tax dollars and the primary beneficiary of biomedical research. However, given the lack of public involvement in research beyond merely enrolling in research, we submit that neither group fully recognize the rights and responsibilities of the public. This is in part due to the fact that to this day, these rights and responsibilities of the community at large as an important key stakeholder in the effort of advancing health through research and discovery have not been carefully examined and delineated. In this paper, we argue that the time has come to investigate the nature of the rights and responsibilities of the public in advancing health through research and discovery. We will argue that the public has the right not merely to participate in research and have their rights protected, but that they have a right to engage in and take ownership in research. In doing so, we seek to begin a dialogue that will include both stakeholders in transformational efforts to improve health through research and discovery.

### Initiatives to accelerate medical research

In 2003 NIH announced a strategy to accelerate medical research which led to the NIH Roadmap for Medical Research. The main goal was, and still is, to accelerate the discovery and delivery of new medical treatments to improve patient care and public health. The thrust of the argument is that “to improve human health, scientific discoveries must be translated into practical applications”. To this end in the early 2000 s NIH put forward one key priority: reengineering the clinical research enterprise by creating Translational Research Centers with the goal to improve health: “The NIH Clinical and Translational Science Awards (CTSA) program enables innovative research teams to speed discovery and advance science aimed at improving our nation’s health”. [2] The first CTSAs were awarded to twelve academic medical centers in September 2006. By 2012, a national consortium of 60 CTSA institutions emerged, all committed to improve human health and enhance the conduct, quality, and dissemination of clinical and translational research.

The second initiative was a new regulatory science initiative outlined by the Food and Drug Administration (FDA) in their 2010 document *Advancing Regulatory Science for Public Health*. [3] The initiative called for a modernization of the evaluative and approval processes to ensure novel products reach patients who need them and to promote and protect the health of the public: “Advances in regulatory science will help make the evaluation and approval process more efficient, helping to deliver safe new products to patients faster and strengthening the ability to monitor product use and improve performance, thus enhancing patient outcomes”. Similar efforts include the 21st Century Cures Act, signed into law in December of 2016 which seeks to accelerate the development of drugs and devices in order to make them available to patients faster, and the revisions to the “Common Rule” by the Office of Human Research Protections, which were undertaken in order to, among other goals, modernize the regulatory system for research in the United States while “facilitating valuable research and reducing burden, delay, and ambiguity…” [4].

Both initiatives, translational research and regulatory science, aim at improving patient care and public health. However, while this approach has paved the way to accelerate research and discovery, it operates under a traditional paradigm that focuses on the advancement of science and generalizable knowledge, the protection of research subjects, the promotion of the well-being of patients, and the promotion of the common good. We of course agree with these goals but argue that the approach will hinder the achievement of these goals by not recognizing and including the rights and responsibilities of the public in these efforts. According to this approach researchers and clinicians are in the “driver’s seat” while the second group of stakeholders, the public, are not included in discussions to develop a roadmap to improve health through research and discovery. To this end, we propose the beginning a bi-directional conversation between researchers and the public that will outline the rights and responsibilities of the public. Specifically, such dialogue, and an ensuing document, will provide the basis to empower (rights) and engage the public (responsibilities) in the conduct of biomedical research. We contend that the public has a right to demand, oversee and participate in the advancement of health through research and discovery. However, as noted above, this right is not commonly recognized by the public which we believe is one, though not the only reason for suboptimal engagement of the community in research enterprise. To be sure, for some communities a history of research abuses and well-deserved mistrust of biomedical research plays a large role as well as exemplified by the Tuskegee Syphilis Study. [5] In concert with other efforts to address mistrust, engaging in bi-directional dialogue around the rights of the public to share ownership in research can help repair this breach in trust, improve enrollment, and ultimately improve the health of our communities through research and discovery.

### Rights and responsibilities

Public investment in the advancement of health through research and discovery is not novel, but the breadth and scope of what we propose is. As we note above, the public in the United States is already financially invested by funding research and discovery at the National Institutes of Health (NIH), National Science Foundation (NSF), Patient Centered Outcomes Research Institute (PCORI), and others. The public is also invested every time they organize grass roots movements that seek to advance health. Consider for example the Pablove Foundation. [6] This foundation, founded by a father who lost his son to a rare childhood cancer, has raised millions of dollars to both fund cutting edge cancer research via seed money to lead eventually to larger grants, including NIH, as well as provide a sense of normalcy to children undergoing cancer treatment with art classes. [7–9] The Pablove foundation is an example of what can happen when the public is invested, not just financially but personally, in advancing health through research and discovery. Furthermore, this example demonstrates that the research community can gain tremendous support when the biomedical research is motivated by compassion and a sense of participation in the promoting of the common good and the improvement of the health of particular communities.

Civil societies function and progress in part because of the rights and their related duties and responsibilities granted to their members. The Bill of Rights, for instance, guarantees some rights (e.g., freedoms of speech, assembly, worship, the right to bear arms, etc.) and are accompanied with responsibilities (e.g., the right to free speech does not protect slander or the shouting fire in a crowded theater). Fundamental to the effectiveness and success of this model is the recognition and exercise of both the rights and the associated responsibilities by the members of a given society. In the context of this paper, we consider the outcome of research and discovery as a social good. The promotion of health ultimately leads to the enhancement of the good of society and consequently the public has a particular interest in setting research priorities and articulating how to achieve particular health outcomes and setting relevant boundaries for biomedical research. To this end, each member of society is a stakeholder and should participate in the effort of advancing health by exercising their rights and fulfilling its responsibilities. Case in point: the recent document published by the Organization for Economic Co-operation and Development (OECD) for responsible innovation of neurotechnology. In their *Recommendation of the Council on Responsible Innovation in Neurotechnology* (2019) the OECD Council made various recommendations aiming at public participation using specifically language such as “enabling societal deliberation”, “promoting cultures of stewardship and trust across the public and private sector”, promoting “a broad public discussion about the best future of neurotechnology in society” to cite a few examples. [10] As discussed by the Council, neurotechnology has great potential to improve human health and innovation in terms of the diagnosis, treatment and prevention of mental and neurological disorders. However, because of the nature of neurotechnology, their ability to collect personal brain data and the centrality of the brain to notions of personal identity, neurotechnology research and its ethical, legal and societal implications must include the input of the public. Such input on the part of the society ought not to be reduced to the collection of data about perceptions of emerging technologies through various surveys. We argue that the public has rights and responsibilities to shape the responsible conduct of research for the advancement of health.

Through this brief analysis we do not seek to determine the basis of these rights but rather to raise awareness that *great* progress in advancing health will require a change in the mindset: from research that strives to advance health as something done by researchers off in their ivory tower academic medical centers to a mindset in which the public sees the advancement of health through research and discovery as their right and partnering in its achievement as their responsibility. We would envision a future in which the public is seen as the stakeholder it ought to be in biomedical research, actively engaged in setting research priorities that matter *to them*. In short, we explicitly put forward the idea that the rights and responsibilities of advancing health through research and discovery is a moral imperative and civic duty that emanates from compassion and basic love for humanity. Specifically, those rights include the right to (1) demand that biomedical research advance health, and (2) partner with other stakeholders to oversee that research not only advances health, but in areas that are of importance to the public. On this view, the public has a right to a seat at the table in priority setting. In parallel public has the responsibility to support, promote and engage in continuum of biomedical research.

We have avoided an in-depth analysis of the nature and scope of these rights and responsibilities in part because the aim of this short paper is a call to bi-directional communication between all stakeholders, but also because we firmly believe that this question is best answered by the stakeholders themselves. These conversations need not wait for a national organized movement, but rather can happen at a local level, facilitated by CTSA’s and their community engagement groups, as one example. Making dramatic progress in improving the health of our communities will require a dramatic shift in how we think about the ownership, engagement, and prioritization of biomedical research to be sure, and it is one that must begin with conversation.

## Data Availability

Not applicable.

## Abbreviations

CTSA: Clinical and Translational Science Awards
FDA: Food and Drug Administration
NIH: National Institutes of Health
NSF: National Science Foundation
OECD: Organization for Economic Co-operation and Development
PCORI: Patient Centered Outcomes Research Institute
